# Pseudotime‐Derived Seven‐Gene Prognostic Signature and TYRP1 in Melanoma

**DOI:** 10.1155/ijog/5922810

**Published:** 2026-11-02

**Authors:** Xiaoqing Li, Yilin Deng, Yue Song, Xiaohui Liu, Hongli Li

**Affiliations:** ^1^ Department of Phase I Clinical Trial Tianjin Medical University Cancer Institute and Hospital Tianjin China tmucih.com

**Keywords:** immune checkpoint, melanoma, prognostic model, single-cell transcriptomics, tumor microenvironment, tumor mutational burden, TYRP1

## Abstract

**Background:**

Melanoma is characterized by high malignancy and complex immune microenvironment heterogeneity. A robust prognostic framework that integrates tumor cell state diversity with clinical outcome remains lacking.

**Methods:**

Integrative scRNA‐seq analysis of public melanoma datasets was performed using Harmony batch correction, unsupervised clustering, and Monocle 3 pseudotime trajectory reconstruction to characterize tumor cell state transitions. Pseudotime‐dynamic genes were subjected to univariate Cox screening across four independent cohorts (GSE19234, GSE22153, GSE65904, TCGA), followed by LASSO‐Cox regression to construct a multigene prognostic model. Validation encompassed immune infiltration estimation, pathway activity scoring, tumor mutational burden (TMB) analysis, and protein‐level expression assessment.

**Results:**

scRNA‐seq identified 14 major cell populations, within which tumor cell reclustering resolved seven functional states: melanocytic, neural crest‐like, MHC‐II high, stress/transitional, proliferating, IFN‐stimulated, and invasive. Pseudotime analysis reconstructed a directional evolution trajectory originating from the melanocytic state, with the stress/transitional state acting as a central hub. Gene module enrichment further delineated the core transcriptional programs of each state. A seven‐gene prognostic model (GBP4, ITGB4, GBP2, TNFSF13B, TSC22D3, CIITA, TYRP1) achieved consistent survival stratification across all four cohorts (all *p* < 0.01) and outperformed 76 published signatures by C‐index ranking. High‐risk patients exhibited an immunosuppressive microenvironment, activation of protumorigenic pathways, and broad suppression of the cancer–immunity cycle. Risk score and TMB were statistically independent, yet their combined stratification significantly refined prognostic resolution (*p* < 0.001). TYRP1, the sole risk gene in the model, demonstrated tumor‐specific protein upregulation by IHC and consistently predicted poor overall survival across three independent transcriptomic cohorts.

**Conclusion:**

This study presents an integrated single‐cell and multicohort prognostic framework for melanoma, revealing the mechanistic link between tumor cell state evolution and immune microenvironment remodeling, and establishing TYRP1 as an independent prognostic biomarker to guide precision immunotherapy decision‐making.

## 1. Introduction

Melanoma represents the most lethal form of skin malignancy and, despite ranking 17th in incidence among all skin cancers, imposes a substantial global disease burden. According to GLOBOCAN 2022, approximately 331,722 new cases and 58,667 deaths are attributed to melanoma annually worldwide, with incidence rates continuing to rise in high Human Development Index regions including Australia, North America, and Europe [1]. Although systemic therapies centered on immune checkpoint inhibitors (ICIs) have markedly improved outcomes in advanced melanoma—the Phase III CheckMate 067 trial demonstrated that nivolumab plus ipilimumab achieved a median overall survival (OS) of 72.1 months at 6.5 years of follow‐up, significantly surpassing monotherapy and chemotherapy [2]—approximately 50% of patients still experience disease progression due to primary or acquired resistance [3]. The mechanistic complexity of therapeutic resistance and the profound interpatient prognostic heterogeneity underscore an urgent need for precise prognostic models with cross‐cohort generalizability, capable of supporting individualized patient stratification and treatment decision‐making.

Intratumoral heterogeneity constitutes the central biological basis underlying variable treatment responses and clinical outcomes in melanoma [4]. Tirosh et al. pioneered the application of single‐cell RNA sequencing (scRNA‐seq) to systematically dissect the multicellular ecosystem of melanoma, revealing extensive transcriptional heterogeneity among malignant cells with respect to cell cycle state, spatial distribution, and drug resistance programs [5]. Building on this foundation, Tsoi et al. subsequently identified four continuous differentiation states in low‐passage melanoma cell lines—melanocytic, transitory, neural crest‐like, and undifferentiated—and demonstrated that these states are differentially associated with ferroptosis sensitivity and pharmacological response [6]. More recently, single‐cell atlas studies of the melanoma tumor microenvironment (TME) have further demonstrated that the composition and functional status of immune cell infiltrates differ markedly between high‐ and low‐risk patients, and that tumor cell state transitions are tightly coupled with immune evasion programs [7]. Nevertheless, existing studies have largely remained at the level of static cell‐type characterization, and a systematic framework integrating pseudotime‐derived dynamic state transitions with multicohort prognostic modeling remains lacking.

Pseudotime trajectory analysis provides a powerful tool for reconstructing the dynamic evolutionary paths of tumor cell states, with the key advantage of extracting biologically meaningful dynamic gene sets from continuous transcriptional transitions to serve as a source of prognostic candidates. Prognostic models built on LASSO‐Cox regularized regression frameworks have demonstrated predictive performance superior to conventional clinical staging systems across multiple solid tumor types; however, existing melanoma prognostic models broadly suffer from single‐cohort validation, unclear biological origins of candidate genes, and the absence of head‐to‐head comparisons between models, all of which limit their translational potential [8].

Tyrosinase‐related protein 1 (TYRP1) is a key enzyme in the melanogenesis pathway, specifically expressed in melanocytes and melanoma cells, with absent expression in normal nonmelanocytic tissues. Early studies indicated that high TYRP1 mRNA expression is significantly associated with shorter distant metastasis‐free survival and OS in melanoma patients, and correlates positively with Breslow tumor thickness [9]. More recently, TYRP1 has been re‐evaluated as a CAR‐T cell therapy target, with its high expression across melanoma subtypes—including immune‐excluded tumors resistant to PD‐1 blockade—further highlighting its dual value as both a therapeutic target and a prognostic biomarker [10]. Nevertheless, systematic multicohort validation of TYRP1 as a core risk gene within a prognostic model framework remains absent.

Against this background, the present study integrated publicly available melanoma scRNA‐seq datasets and applied Monocle 3 pseudotime trajectory analysis to systematically identify dynamic gene sets associated with tumor cell state transitions. These genes were subsequently used as a candidate pool, from which 24 prognostically consistent genes were selected by univariate Cox regression across four independent cohorts, and further refined to a seven‐gene prognostic risk score model via LASSO‐Cox regularized regression. The model demonstrated robust survival stratification across all four cohorts and confirmed its competitive advantage through head‐to‐head comparison with 76 published melanoma prognostic signatures. Furthermore, the biological underpinnings of the model were systematically elucidated from multiple dimensions, encompassing immune microenvironment remodeling, tumor mutational burden (TMB) association, and cancer–immunity cycle suppression. TYRP1, the sole risk gene within the model, was independently validated at both the protein expression and multicohort transcriptomic levels. This study provides a novel molecular tool and theoretical rationale for precise prognostic stratification and immunotherapy candidate selection in melanoma.

## 2. Method

### 2.1. scRNA‐seq Data Acquisition and Quality Control

scRNA‐seq data were obtained from the Gene Expression Omnibus (GEO) database (Accession: GSE277165 [11]), encompassing tumor tissue samples from multiple primary melanoma patients, comprising 10 primary melanoma tumor samples from 10 patients. Quality control was performed using Seurat [12]. Cells with mitochondrial gene fraction > 20%, fewer than 200 or more than 6000 detected genes, or putative doublets were excluded. Retained cells were normalized using NormalizeData() (scale factor = 10,000, log‐transformed), and highly variable genes were identified via FindVariableFeatures() (nfeatures = 2000) for downstream dimensionality reduction.

### 2.2. Batch Correction, Clustering, and Cell Type Annotation

Batch effects across samples were corrected using Harmony [13] applied to the Top 30 principal components (PCs). A shared nearest‐neighbor (SNN) graph was constructed with FindNeighbors() (dims = 1 : 30), followed by unsupervised clustering via FindClusters() (Leiden algorithm, resolution = 0.5). This resolution was selected to balance cluster stability and separation while recovering the expected marker‐defined cell populations. Two‐dimensional visualization was performed with UMAP (RunUMAP(), dims = 1 : 30). Cluster marker genes were identified using FindAllMarkers() (Wilcoxon rank‐sum test, min.pct = 0.25, log2FC threshold = 0.25, Bonferroni correction). Cell types were annotated based on canonical marker genes from published melanoma single‐cell atlases, including MLANA, S100B, PMEL (tumor cells), ACTA2 (cancer‐associated fibroblasts [CAFs]), CD3D (T cells), LYZ (macrophages), MS4A1 (B cells), NKG7 (NK/NKT cells), PECAM1 (endothelial cells), KRT14 (keratinocytes), TPSB2 (mast cells), and MKI67 (proliferating cells).

### 2.3. Tumor Cell Subclustering and Functional State Annotation

Tumor cells were subsetted and reclustered following reselection of highly variable genes, principal component analysis (PCA), Harmony correction, and UMAP embedding at higher resolution (resolution = 0.3). Seven functional states were identified and annotated by reference to published melanoma tumor cell state classification frameworks: melanocytic, neural crest‐like, MHC‐II high, stress/transitional, proliferating, IFN‐stimulated, and invasive. Marker gene expression was visualized using DotPlot() (dot size = percentage of expressing cells; color = z − score − normalized mean expression) and FeaturePlot().

### 2.4. Pseudotime Trajectory Analysis

Pseudotime analysis was performed using Monocle 3 [14]. The Seurat object was converted to CellDataSet format, and cell state transition trajectories were inferred using learn_graph() with UMAP coordinates as the embedding. The Melanocytic cluster was designated as the root node, and pseudotime values were computed with order_cells(). Pseudotime was visualized on UMAP with a continuous color scale, and distributions across tumor states were displayed as violin plots (ggplot2).

### 2.5. Gene Module Identification and Hallmark Pathway Enrichment

Genes exhibiting significant pseudotime‐dependent dynamics were identified using graph_test() (Moran′s I test, *q* < 0.05), with *q* < 0.05 defined a priori as the Benjamini–Hochberg false–discovery‐rate threshold in Monocle 3 and grouped into coexpression modules via find_gene_modules() (Louvain clustering, resolution = 1e − 2). Module activity scores per tumor state were z‐score normalized and displayed as a heatmap. Over‐representation analysis (ORA) for each module was performed with enricher() in clusterProfiler against the hallmark gene sets (50 pathways, MSigDB H collection) [15–17] retrieved via msigdbr. *p* values were adjusted using the Benjamini–Hochberg method, and results were plotted as bar charts with –log₁₀(FDR) on the *x*‐axis (dashed line: FDR = 0.05).

### 2.6. Multicohort Data Integration and Batch Effect Correction

Three independent bulk gene‐expression datasets from the GEO repository (GSE19234 [18], GSE22153 [19], GSE65904 [20, 21]) were downloaded and combined with The Cancer Genome Atlas Skin Cutaneous Melanoma cohort (TCGA‐SKCM), accessed through the Genomic Data Commons (GDC; https://portal.gdc.cancer.gov/projects/TCGA-SKCM) [22, 23] to establish a multicohort validation framework. Expression matrices from each cohort were normalized to log2(TPM + 1), followed by batch effect removal using the removeBatchEffect() function from the limma package. Correction efficacy was assessed by PCA, with intercohort sample distribution evaluated by the variance explained by Dim1 and Dim2.

### 2.7. Identification of Prognostic Genes

Dynamically expressed genes identified along the pseudotime trajectory were used as the candidate gene set. Univariate Cox proportional hazards regression was performed independently in each cohort with OS as the endpoint. Only genes reaching statistical significance (*p* < 0.05) in at least two cohorts were retained. Chromosomal distribution of these genes was visualized using a circos plot. A multicohort forest plot summarized the hazard ratios (HR) and 95% confidence intervals across all four cohorts; HR > 1 denoted risk genes and HR < 1 denoted protective genes.

### 2.8. Copy Number Variation Analysis

Somatic copy number alteration (SCNA) data from the TCGA cohort were used to calculate CNV frequencies for each prognostic gene. Amplification (AMP) and deletion (DEL) rates were displayed as stacked bar charts, grouped by chromosomal location.

### 2.9. LASSO‐Cox Prognostic Model Construction

Prognostic genes identified through multicohort screening were subjected to LASSO‐penalized Cox regression using the glmnet package. The optimal regularization parameter *λ* was determined by minimizing the partial likelihood deviance through 10‐fold cross‐validation (lambda.min = 0.023). Genes with nonzero coefficients at the optimal *λ* were retained in the final model, with their coefficients visualized by a bar chart and the full regularization path displayed as a coefficient trace plot. In summary, the complete model‐development workflow proceeded as follows: pseudotime‐dynamic genes → 24 genes significant by univariate Cox regression (*p* < 0.05) in at least two of the four cohorts → LASSO‐Cox regression with 10‐fold cross‐validation (lambda.min = 0.023), yielding seven nonzero‐coefficient genes.

### 2.10. Prognostic Model Validation and Performance Evaluation

Risk scores were calculated for each patient by summing the product of each model gene′s regression coefficient and its standardized expression level across the seven LASSO‐Cox–selected genes (GBP4, ITGB4, GBP2, TNFSF13B, TSC22D3, CIITA, TYRP1). Patients were stratified into high‐ and low‐risk groups using the median risk score of each cohort as the cutoff. Kaplan–Meier survival curves were generated and group differences were assessed by the log‐rank test. Time‐dependent ROC curves and AUC values at 1, 2/3, and 5 years were computed using the R package timeROC. Specifically, AUCs were estimated at 1, 3, and 5 years in TCGA and at 1, 2, and 3 years in the GEO cohorts, reflecting differences in follow‐up duration; horizons were selected to ensure adequate numbers at risk and events at each time point. All analyses were performed independently in four cohorts: TCGA (*N* = 469), GSE19234 (*N* = 44), GSE22153 (*N* = 54), and GSE65904 (*N* = 210). Prognostic gene signatures reported in published melanoma studies were retrieved, and their C‐indices were recalculated in each cohort using the concordance() function from the R package survival for head‐to‐head comparison with the present model. Additionally, standardized expression matrices of the seven model genes were extracted and subjected to PCA using prcomp(); PC1–PC2 scatter plots were colored by risk group.

### 2.11. Multidimensional Quantification of the Tumor Immune Microenvironment (TIME)

To systematically evaluate the relationship between the prognostic risk model and the TIME, we conducted analyses across three complementary dimensions: immune cell infiltration quantification, differential expression of immune‐related functional gene sets, and microenvironmental scoring. Immune infiltration was estimated by integrating seven established algorithms—TIMER, CIBERSORT, CIBERSORT‐ABS, QUANTISEQ, MCPCOUNTER, XCELL, and EPIC—implemented uniformly through the R package IOBR [24]. Immune cell abundance was estimated for high‐ and low‐risk groups within the TCGA‐SKCM cohort and visualized as a heatmap. Differential expression of immune‐related functional gene sets was assessed across five categories: MHC Class I molecules (B2M, HLA‐A, HLA‐B, HLA‐C, TAP1, TAP2), MHC Class II molecules (HLA‐DPA1, HLA‐DPB1, HLA‐DPB2, HLA‐DQA1, HLA‐DQA2, HLA‐DQB1, HLA‐DQB2, HLA‐DRB1, HLA‐DRB5, HLA‐DRB6), other MHC molecules (HLA‐E, HLA‐F, HLA‐G, HLA‐H), coinhibitory molecules (BTLA, BTN1A1, BTN2A2, BTN3A1, BTNL2, CD274, CD48, CTLA4, HAVCR2, LAG3, LAIR1, PDCD1, PDCD1LG2, TIGIT, VTCN1), and costimulatory molecules (BTNL8, CD226, CD27, CD28, CD58, CD70, CD80, CD86, HAVCR1, ICOS, ICOSLG, SLAMF1, TMIGD2, and members of the TNFRSF/TNFSF family). Between‐group differences were evaluated using the Wilcoxon rank‐sum test, with *p* < 0.05 considered statistically significant. Microenvironmental scores—StromaScore, ImmuneScore, ESTIMATEScore, and TumorPurity—were computed by the ESTIMATE algorithm from gene expression profiles, and their associations with the risk score were quantified by Spearman correlation analysis; a Benjamini–Hochberg‐adjusted *q* < 0.05 was considered statistically significant.

### 2.12. Integrative Analysis of the Prognostic Model With TMB and Immune‐Related Pathways

To further characterize the biological underpinnings of the prognostic risk model, multidimensional functional analyses were performed using TCGA melanoma gene expression and somatic mutation data. Gene set variation analysis (GSVA) was applied to quantify pathway activity scores for the seven steps of the cancer–immunity cycle (Steps 1–7: cancer antigen release, presentation, priming and activation, immune cell recruiting, T cell infiltration into tumors, recognition of cancer cells by T cells, and killing of cancer cells) and 17 cancer‐related pathways (including IFN‐Gamma_signature, APM_signal, Cell_cycle, DNA_replication, Mismatch_repair, Nucleotide_excision_repair, Homologous_recombination, Fanconi_anemia_pathway, p53_signaling_pathway, Proteasome, Spliceosome, MicroRNAs_in_cancer, Base_excision_repair, Oocyte_meiosis, Progesterone‐mediated_oocyte_maturation, Pyrimidine_metabolism, and Viral_carcinogenesis). Spearman correlation analysis was used to construct a pathway–immunity cycle correlation network, in which line thickness encodes correlation coefficient magnitude and solid/dashed lines denote positive/negative correlations; significance threshold was set at *q* < 0.001. Somatic mutation data in MAF format were processed using the maftools package to generate waterfall plots depicting the Top 20 most frequently mutated genes in high‐ and low‐risk groups, with flanking bar charts summarizing mutations by risk group. TMB was defined as the total number of nonsynonymous somatic mutations per megabase. Group differences in standardized TMB were assessed by Wilcoxon rank‐sum test. The correlation between risk score and TMB was evaluated by Spearman analysis. Patients were further stratified into four subgroups by the median risk score and median TMB (H‐TMB + high risk, H‐TMB + low risk, L‐TMB + high risk, L‐TMB + low risk), and Kaplan–Meier survival curves were generated with group differences evaluated by the log‐rank test.

### 2.13. Validation of TYRP1 Protein Expression and Multicohort Survival Analysis

Immunohistochemical (IHC) staining data for TYRP1 in normal skin tissue and melanoma tissue were retrieved from the Human Protein Atlas (HPA) database to assess protein expression levels, staining intensity, and subcellular localization. To further validate the prognostic value of TYRP1, patients in three independent cohorts (TCGA, *N* = 469; GSE19234, *N* = 44; GSE65904, *N* = 210) were stratified into high‐ and low‐expression groups based on the median TYRP1 expression level within each cohort. Kaplan–Meier survival curves were generated and between‐group differences were evaluated by the log‐rank test.

## 3. Result

### 3.1. Single‐Cell Transcriptomic Profiling Reveals the Cellular Composition of the Melanoma TME

To comprehensively characterize the cellular composition and transcriptional heterogeneity of the melanoma TME at single‐cell resolution, we performed integrative scRNA‐seq analysis of publicly available melanoma datasets. Following stringent quality control, library‐size normalization, and Harmony‐based batch correction, unsupervised clustering and UMAP visualization identified 14 major cell populations encompassing the core cellular constituents of the melanoma TME (Figure 1A). Each cluster was annotated based on the differential expression of canonical marker genes (Figure 1B): MLANA, S100B, and PMEL specifically marked tumor cells; ACTA2 marked CAFs; CD3D marked T cells; LYZ marked macrophages; MS4A1 marked B cells; NKG7 marked NK/NKT cells; PECAM1 marked endothelial cells; KRT14 marked keratinocytes; TPSB2 marked mast cells; and MKI67 was enriched in proliferating cells. The concordance between marker expression patterns and cluster annotations confirmed the robustness of the cell type classification.

**Figure 1:**
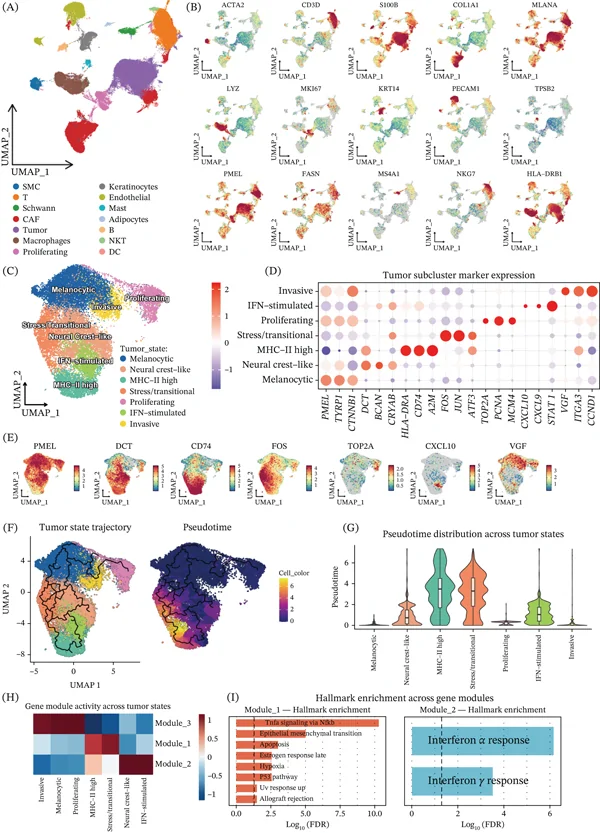
Single‐cell transcriptomic landscape and tumor cell state heterogeneity in melanoma. (A) UMAP visualization of all single cells from melanoma samples following Harmony batch correction, with 14 major cell populations indicated by distinct colors. (B) Feature plots showing the UMAP expression distribution of canonical cell‐type marker genes. Color intensity reflects log‐normalized expression. (C) UMAP of subclustered tumor cells, revealing seven functional states distinguished by color. (D) Dot plot of state‐specific marker genes across the seven tumor functional states. Dot size denotes the fraction of expressing cells; color represents z‐score–normalized mean expression. (E) Feature plots of representative marker genes (PMEL, DCT, CD74, FOS, TOP2A, CXCL10, VGF) projected onto the tumor cell UMAP. (F) Pseudotime trajectory inferred by Monocle 3. Left: cells colored by functional state with principal graph overlaid (black curves). Right: cells colored by pseudotime value (dark purple to yellow = low to high). (G) Violin plots showing pseudotime value distributions across tumor functional states. (H) Heatmap of z‐score–normalized mean module activity scores for three gene modules (Module_1–3) across tumor functional states. (I) Bar plots of hallmark over‐representation analysis (ORA) results for Module_1 (left) and Module_2 (right). *x*‐axis: –log₁₀(FDR); dashed line: FDR = 0.05. *p* values adjusted by Benjamini–Hochberg correction. Source and figure preparation: Panels A–I were created by the authors specifically for this manuscript using R Version 4.4.2, based on single‐cell RNA‐sequencing data obtained from the Gene Expression Omnibus (GEO), accession GSE277165 (https://www.ncbi.nlm.nih.gov/geo/query/acc.cgi?acc=GSE277165). Analyses and visualizations used Seurat, Harmony, Monocle 3, ggplot2, clusterProfiler, and msigdbr, as described in the Methods. Hallmark gene sets used in Panel I were obtained from the Molecular Signatures Database (MSigDB; https://www.gsea-msigdb.org/gsea/msigdb) [15–17]. The panels present author‐generated analyses of public data rather than figures reproduced from the original dataset publications.

### 3.2. Tumor Cell Subclustering Delineates Seven Distinct Functional States

To resolve intratumoral transcriptional heterogeneity, tumor cells were independently reclustered, yielding seven functionally distinct states (Figure 1C). Marker gene analysis revealed state‐specific transcriptional signatures (Figure 1D): the melanocytic state was characterized by high expression of melanin synthesis genes PMEL, TYRP1, DCT, and CTNNB1, indicative of a well‐differentiated phenotype; the neural crest‐like state specifically expressed BCAN and CRYAB, reflecting dedifferentiation toward a neural crest progenitor identity; the MHC‐II high state was enriched for antigen presentation genes HLA‐DRA, CD74, and A2M, suggesting enhanced immunomodulatory capacity; the stress/transitional state exhibited upregulation of stress‐responsive transcription factors FOS, JUN, and ATF3; the proliferating state was defined by cell cycle genes TOP2A, PCNA, and MCM4; the IFN‐stimulated state showed elevated expression of interferon response genes CXCL9, CXCL10, and STAT1; and the invasive state specifically upregulated VGF, ITGA3, and CCND1, consistent with enhanced migratory and invasive potential. These annotations were further validated by representative marker gene expression maps on UMAP (Figure 1E).

### 3.3. Pseudotime Trajectory Analysis Reconstructs the Dynamic Evolution of Tumor Cell States

Pseudotime analysis using Monocle 3 reconstructed the continuous state transition trajectory of tumor cells (Figure 1F). Using the melanocytic cluster as the root, pseudotime distributions across states revealed a directional progression (Figure 1G). Melanocytic cells were concentrated at near‐zero pseudotime values, consistent with a highly differentiated, stable baseline state. Neural crest‐like cells showed progressively elevated pseudotime, representing early dedifferentiation. The stress/transitional state exhibited the broadest pseudotime distribution, positioning it as a pivotal transitional hub bridging multiple cell states. MHC‐II high cells displayed the highest median pseudotime, occupying the terminal end of the main trajectory. Notably, proliferating, IFN‐stimulated, and invasive states clustered at low pseudotime values and occupied distinct UMAP positions, suggesting that these states represent independent functional branches driven by specific microenvironmental cues rather than sequential nodes along the main differentiation axis.

### 3.4. Gene Module and Pathway Enrichment Analyses Uncover Core Transcriptional Programs Underlying State Transitions

Weighted coexpression analysis of pseudotime‐dynamic genes identified three tumor‐state‐specific gene modules (Figure 1H). Module_1 was most active in invasive and melanocytic states and was significantly enriched for TNF*α* Signaling via NF‐*κ*B, epithelial‐mesenchymal transition (EMT), apoptosis, hypoxia, p53 pathway, and UV response (Figure 1I), collectively representing a core transcriptional program associated with late‐stage invasive adaptation and stress resilience. Module_2 was selectively activated in the IFN‐stimulated state and exclusively enriched for interferon alpha response and interferon gamma response pathways (Figure 1I), demonstrating that sustained interferon signaling from the immune microenvironment is a key upstream driver of this distinct transcriptional state. Together, these findings systematically delineate the multilayered transcriptional regulatory mechanisms governing melanoma tumor cell state evolution and their functional underpinnings.

### 3.5. Multicohort Prognostic Gene Screening and LASSO‐Cox Model Construction

To build a robust prognostic model with cross‐cohort generalizability, we integrated four independent melanoma cohorts—GSE19234, GSE22153, GSE65904, and TCGA. After batch effect correction, PCA demonstrated uniform distribution of samples across cohorts in the reduced‐dimensional space (Dim1 = 10.9*%*, Dim2 = 8.6*%*), confirming the quality of data integration (Figure 2A). Using pseudotime‐derived dynamic genes as candidates, univariate Cox regression across the four cohorts identified 24 genes with consistent prognostic associations, whose chromosomal distribution is displayed in a circos plot (Figure 2B). The multicohort forest plot revealed that the majority of these genes exerted protective effects (HR < 1), with TYRP1 being the only significant risk gene (HR > 1) (Figure 2C). CNV analysis showed that genes on Chromosome 6, including multiple HLA family members, exhibited frequent AMP, whereas TYRP1 on Chromosome 9 was predominantly subject to DEL, suggesting that copy number alterations may underlie the dysregulated expression of these prognostic genes (Figure 2D). LASSO‐Cox regression further refined the 24 candidates to 7 genes with nonzero coefficients at the optimal *λ* = 0.023 (GBP4, ITGB4, GBP2, TNFSF13B, TSC22D3, CIITA, TYRP1) (Figure 2E,F). Tenfold cross‐validation confirmed that this *λ* value corresponded to the minimum prediction error, demonstrating stable model performance (Figure 2G), thereby laying the foundation for subsequent risk score model construction.

**Figure 2:**
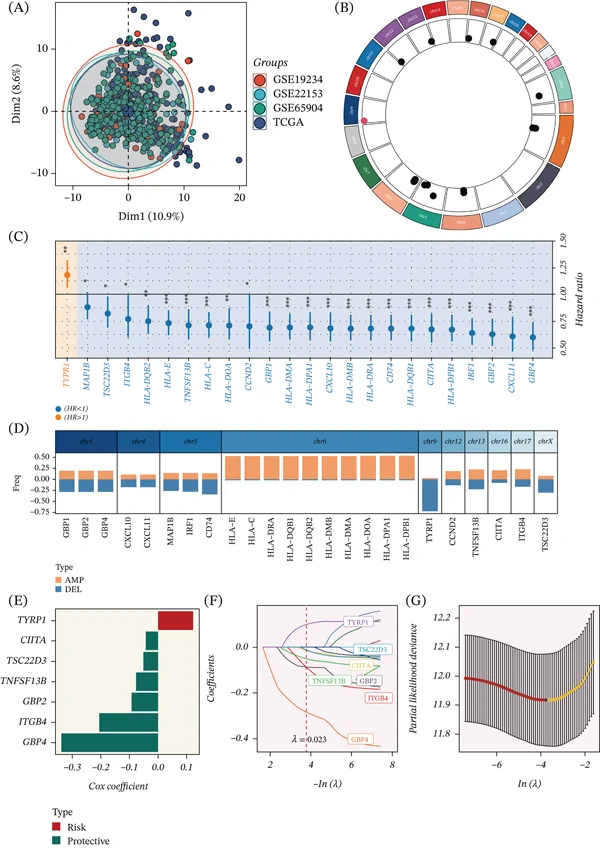
Multicohort prognostic gene screening and LASSO‐Cox predictive model construction. (A) PCA plot of four cohorts after batch effect correction; ellipses indicate 95% confidence regions per cohort. (B) Circos plot of chromosomal distribution of prognostic genes; black dots mark significant gene positions. (C) Multicohort Cox regression forest plot for 24 prognostic genes; points indicate HR, bars indicate 95% CI; orange, risk genes (HR > 1); blue, protective genes (HR < 1).  ^∗^ *p* < 0.05,  ^∗∗^ *p* < 0.01,  ^∗∗∗^ *p* < 0.001. (D) CNV frequency bar chart; orange, amplification (AMP); blue, deletion (DEL); grouped by chromosome. (E) LASSO‐selected gene coefficients in the Cox model; red, risk factors; green, protective factors. (F) LASSO regularization path; dashed line marks the optimal *λ* = 0.023. (G) Tenfold cross‐validation deviance curve; *y*‐axis, partial likelihood deviance; vertical bars, standard error. Source and figure preparation: Panels A–G were created by the authors specifically for this manuscript using R Version 4.4.2. Bulk expression and clinical data were obtained from GEO datasets GSE19234 (https://www.ncbi.nlm.nih.gov/geo/query/acc.cgi?acc=GSE19234), GSE22153 (https://www.ncbi.nlm.nih.gov/geo/query/acc.cgi?acc=GSE22153), GSE65904 (https://www.ncbi.nlm.nih.gov/geo/query/acc.cgi?acc=GSE65904) [20, 21], and from The Cancer Genome Atlas Skin Cutaneous Melanoma project (TCGA‐SKCM), accessed through the Genomic Data Commons (GDC; https://portal.gdc.cancer.gov/projects/TCGA-SKCM) [22, 23]. Copy‐number alteration data in Panel D were derived from TCGA‐SKCM. Analyses included batch‐effect correction using limma and LASSO‐Cox modeling using glmnet. All panels display analyses performed by the authors for this study.

### 3.6. The Prognostic Model Demonstrates Robust Survival Stratification and Predictive Performance Across Independent Cohorts

To systematically evaluate the generalizability of the constructed prognostic model, we performed comprehensive validation across four independent melanoma cohorts. Kaplan–Meier survival analysis consistently showed that patients in the high‐risk group had significantly shorter OS than those in the low‐risk group in all four cohorts (all *p* < 0.01), indicating stable patient stratification capability (Figure 3A–D). Time‐dependent ROC analysis further confirmed the model′s predictive performance: AUC values in the TCGA cohort were 0.62, 0.66, and 0.67 at 1, 3, and 5 years, respectively; in GSE19234, AUC values reached 0.93, 0.81, and 0.91 at 1, 2, and 3 years, representing the strongest performance among all cohorts; in GSE22153, AUC values were 0.81, 0.82, and 0.76 at 1, 2, and 3 years; and in GSE65904, AUC values were 0.65, 0.68, and 0.67 at 1, 2, and 3 years. Head‐to‐head comparison of C‐indices with 76 published melanoma prognostic models revealed that the present model (My model) ranked among the top performers in all four cohorts, outperforming the majority of existing signatures (Figure 3E–H). PCA based on the expression profiles of the seven model genes further demonstrated clear separation between high‐ and low‐risk patients in PC1–PC2 space across all cohorts (Figure 3I–L), providing transcriptome‐level independent validation of effective patient stratification and confirming the biological robustness and potential clinical utility of this prognostic model.

Figure 3Multicohort validation and predictive performance of the prognostic model. (A–D) Kaplan–Meier survival curves (left) and time‐dependent ROC curves (right) based on risk scores in the TCGA (*N* = 469), GSE19234 (*N* = 44), GSE22153 (*N* = 54), and GSE65904 (*N* = 210) cohorts. Red, high‐risk group; green, low‐risk group. *p* values were determined by the log‐rank test. (E–H) C‐index comparison between the present model (My model) and published melanoma prognostic signatures across four cohorts. (I–L) PCA scatter plots based on the expression of seven model genes, showing the distribution of high‐risk (red) and low‐risk (green) patients in PC1–PC2 space. Source and figure preparation: Panels A–L were created by the authors specifically for this manuscript using R Version 4.4.2. Data sources were The Cancer Genome Atlas Skin Cutaneous Melanoma project (TCGA‐SKCM), accessed through the Genomic Data Commons (GDC; https://portal.gdc.cancer.gov/projects/TCGA-SKCM) [22, 23], and GEO datasets GSE19234 (https://www.ncbi.nlm.nih.gov/geo/query/acc.cgi?acc=GSE19234), GSE22153 (https://www.ncbi.nlm.nih.gov/geo/query/acc.cgi?acc=GSE22153), GSE65904 (https://www.ncbi.nlm.nih.gov/geo/query/acc.cgi?acc=GSE65904) [20, 21]. Survival and predictive‐performance analyses used survival and timeROC; principal component analysis used the R function prcomp(). The C‐index comparisons in Panels E–H were calculated by the authors using published prognostic signatures; the displayed plots were generated for this study and were not reproduced from those publications.

**Figure:**
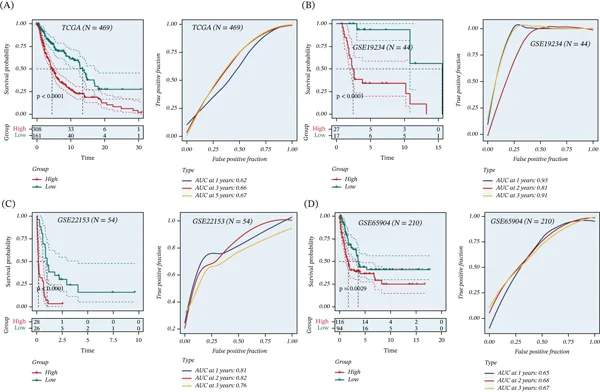

**Figure:**
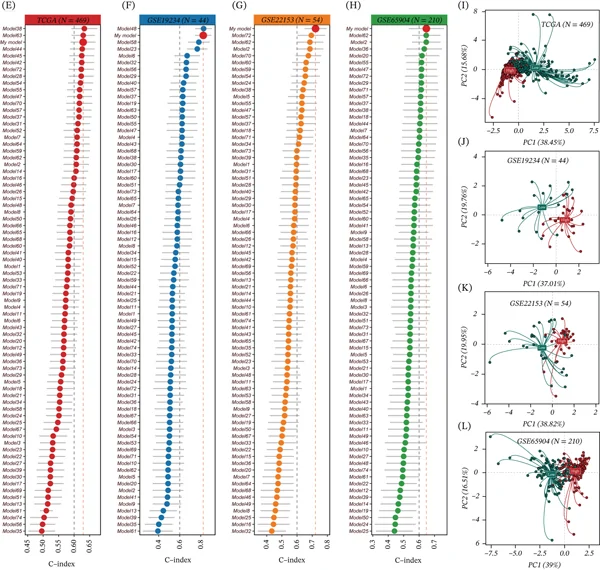

### 3.7. The Prognostic Risk Model Is Closely Associated With the Activation State of the TIME

To determine whether the biological information captured by the prognostic model reflects the immune contexture of the TME, we integrated immune infiltration estimates from seven quantification algorithms (Figure 4A). Across virtually all algorithmic frameworks, low‐risk patients exhibited markedly higher immune cell infiltration, encompassing effector subsets including CD4+ T cells, CD8+ T cells, B cells, NK cells, and dendritic cells, consistent with an immune‐inflamed (“hot tumor”) phenotype. In contrast, high‐risk tumors were characterized by globally depleted immune infiltration, indicative of an immune‐excluded or “cold tumor” state. At the level of immune functional gene expression, low‐risk patients showed broad and significant upregulation of MHC Classes I and II antigen‐presentation molecules, coinhibitory checkpoint molecules—including HAVCR2, LAG3, PDCD1, TIGIT, and CTLA4—as well as the majority of costimulatory molecules (Figure 4B), a transcriptional pattern consistent with an activated adaptive immune response. Notably, VTCN1 and TNFSF9 did not reach statistical significance between the two groups (ns), suggesting that these genes may not be primary effector molecules within the risk stratification framework defined by this model. At the level of global microenvironmental scoring, the risk score was significantly and negatively correlated with StromaScore (*r* = −0.38, *q* = 0), ImmuneScore (*r* = −0.55, *q* = 0), and ESTIMATEScore (*r* = −0.52, *q* = 0), while positively correlated with TumorPurity (*r* = 0.53, *q* = 0) (Figure 4C). These findings indicate that higher risk patients harbor tumors with greater cellular purity, reduced immune and stromal cell infiltration, and an overall immunosuppressive microenvironment. Taken together, the three‐dimensional analysis demonstrates a high degree of concordance between the prognostic risk model and the immune activation state of the TME, suggesting that low‐risk patients may derive greater benefit from immune checkpoint blockade therapy.

**Figure 4:**
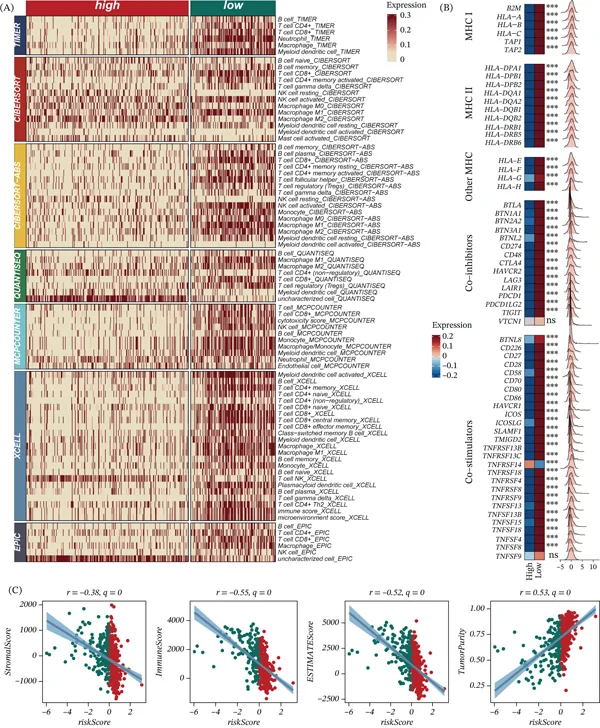
Systematic association between the prognostic risk model and the tumor immune microenvironment. (A) Heatmap of immune cell infiltration abundance estimated by seven algorithms (TIMER, CIBERSORT, CIBERSORT‐ABS, QUANTISEQ, MCPCOUNTER, XCELL, and EPIC). Rows represent immune cell subtypes derived from each algorithm; columns represent TCGA‐SKCM patient samples stratified into high‐risk (left, red header) and low‐risk (right, teal header) groups. Color intensity reflects normalized infiltration abundance (Expression 0–0.3), with dark red indicating high infiltration. (B) Differential expression heatmap and distribution comparison of immune‐related functional gene sets between high‐ and low‐risk groups. The left heatmap displays expression levels of five gene categories—MHC Class I, MHC Class II, other MHC molecules, coinhibitors, and costimulators (blue: low expression; red: high expression). The right panel shows corresponding ridge density plots comparing expression distributions between high‐risk (high) and low‐risk (low) groups. Statistical significance was assessed by the Wilcoxon rank‐sum test:  ^∗∗∗^ *p* < 0.001,  ^∗∗^ *p* < 0.01,  ^∗^ *p* < 0.05; ns, not significant. (C) Spearman correlation scatter plots between the risk score and four ESTIMATE‐derived microenvironmental indices. The *x*‐axis represents the risk score (riskScore); the *y*‐axis represents StromaScore, ImmuneScore, ESTIMATEScore, and TumorPurity, respectively. Green dots indicate low‐risk patients; red dots indicate high‐risk patients. The blue line represents the linear regression fit with the shaded region denoting the 95% confidence interval. Spearman correlation coefficients (r) and Benjamini–Hochberg‐adjusted *q* values are indicated above each plot. Source and figure preparation: Panels A–C were created by the authors specifically for this manuscript using R Version 4.4.2, based on data from The Cancer Genome Atlas Skin Cutaneous Melanoma project (TCGA‐SKCM), accessed through the Genomic Data Commons (GDC; https://portal.gdc.cancer.gov/projects/TCGA-SKCM) [22, 23]. Immune‐cell infiltration estimates were computed using the algorithms implemented through IOBR, and stromal, immune, ESTIMATE, and tumor‐purity scores were calculated using ESTIMATE, as described in the Methods. All panels represent author‐generated analyses and visualizations of these public data.

### 3.8. Association of the Risk Model With TMB and Immune Pathway Activity

To elucidate the biological mechanisms underlying the prognostic model, we systematically examined the relationship between risk scores and both cancer‐related pathway activity and the cancer–immunity cycle (Figure 5A). Risk scores were significantly positively correlated with the majority of cancer‐related pathways—including Cell_cycle, DNA_replication, Mismatch_repair, proteasome, and spliceosome—while showing prominent negative correlations with all seven steps of the cancer—immunity cycle, particularly those governing T cell recruiting, infiltration, recognition, and killing. This pattern indicates that high‐risk patients exhibit concurrent activation of protumorigenic proliferative pathways and systemic suppression of antitumor immune circuitry. Mutation landscape analysis revealed that TTN (67%), MUC16 (62%), and BRAF (51%) were the three most frequently mutated genes, with high‐risk patients displaying a globally higher mutation frequency (Figure 5B). The lower block of Figure 5B displays copy‐number alterations in the seven genes included in the prognostic model. Despite a marginally higher TMB in the high‐risk group, the difference was not statistically significant (*p* = 0.21, Figure 5C), and no meaningful linear association was detected between risk score and TMB (*R* = 0.033, *p* = 0.49, Figure 5D), indicating that the prognostic information captured by the risk score is independent of TMB. When patients were jointly stratified by median risk score and median TMB (Figure 5E), the H‐TMB + low risk group achieved the best OS, whereas the L‐TMB + high risk group had the worst prognosis; the four‐group difference was highly significant (*p* < 0.001). This combined stratification strategy demonstrated superior prognostic resolution compared with either variable alone, offering a refined framework for individualized immunotherapy decision‐making in melanoma. These findings provide a basis for exploratory risk stratification in retrospective cohorts, and their clinical applicability warrants validation in prospective cohorts with immunotherapy treatment and outcome data.

**Figure 5:**
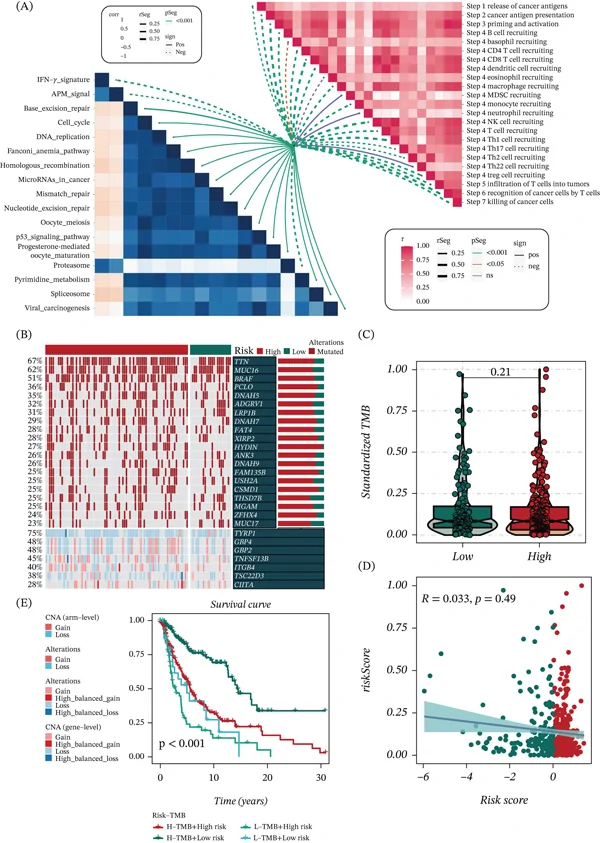
Integrative analysis of the prognostic model with tumor mutational burden and immune‐related pathways. (A) Spearman correlation network between 17 cancer‐related pathways and the seven steps of the cancer–immunity cycle. Cancer‐related pathways are listed on the left; immunity cycle Steps 1–7 are listed on the right. Line color indicates significance level (green: *q* < 0.001; orange: *q* < 0.05; purple: ns), line thickness reflects the magnitude of the correlation coefficient, solid lines denote positive correlations, and dashed lines denote negative correlations. The lower‐left heatmap displays correlations among cancer‐related pathways, and the upper right heatmap displays correlations among cancer–immunity cycle activities, including the immune‐cell recruitment components of Step 4. (B) Genomic alteration landscape in high‐ and low‐risk patients. Each column represents one patient (red header: high‐risk; green header: low‐risk). The upper block shows the 20 most frequently mutated genes, with dark red cells indicating mutation events and percentages on the left indicating mutation frequencies. The flanking bars summarize mutations by risk group. The lower block displays copy‐number alterations in the seven model genes, with alteration categories indicated by the color keys. (C) Violin plot comparing standardized TMB between high‐ and low‐risk groups. Green indicates low‐risk and red indicates high‐risk patients; embedded boxplots display median and interquartile range; individual dots represent single patients. Group difference was assessed by Wilcoxon rank‐sum test (*p* = 0.21). (D) Spearman correlation scatter plot between risk score and standardized TMB. The *x*‐axis represents the risk score and the *y*‐axis represents standardized TMB; green and red dots indicate low‐ and high‐risk patients, respectively; the shaded area represents the 95% confidence interval. *R* = 0.033, *p* = 0.49. (E) Kaplan–Meier survival curves for patients jointly stratified by risk score and TMB. Patients were dichotomized by the median risk score and median TMB and divided into four groups: H‐TMB + high risk (red), H‐TMB + low risk (dark green), L‐TMB + high risk (teal), and L‐TMB + low risk (light blue). The *x*‐axis represents follow‐up time (years) and the *y*‐axis represents overall survival probability; tick marks indicate censored events. Log‐rank test *p* < 0.001. Source and figure preparation: Panels A–E were created by the authors specifically for this manuscript using R Version 4.4.2. Gene‐expression, somatic‐mutation, and clinical data were obtained from The Cancer Genome Atlas Skin Cutaneous Melanoma project (TCGA‐SKCM), accessed through the Genomic Data Commons (GDC; https://portal.gdc.cancer.gov/projects/TCGA-SKCM) [22, 23]. Pathway activity was assessed using GSVA, and somatic‐mutation data were processed using maftools, as described in the Methods. All panels display analyses and visualizations generated by the authors for this study.

### 3.9. Protein Expression and Prognostic Validation of TYRP1 as the Sole Risk Gene

Given its designation as the sole risk gene in the prognostic model, TYRP1 was further characterized at the protein and transcriptomic levels. IHC images retrieved from the HPA database revealed a striking contrast in TYRP1 expression between normal and malignant tissue: staining was entirely absent in epidermal cells of normal vulvar skin (Figure 6A), whereas melanoma tumor cells displayed strong, diffuse positivity with cytoplasmic and membranous localization (Figure 6B), pointing to tumor‐specific protein upregulation. This expression pattern was mirrored at the transcriptomic level across three independent cohorts. In TCGA (*N* = 469), GSE19234 (*N* = 44), and GSE65904 (*N* = 210), patients with high TYRP1 expression uniformly exhibited significantly shorter OS compared with the low‐expression group (log‐rank *p* < 0.0001, *p* = 0.00014, and *p* = 0.008, respectively; Figure 6C–E). Together, these convergent lines of evidence position TYRP1 as a robust and independently validated prognostic biomarker in melanoma.

**Figure 6:**
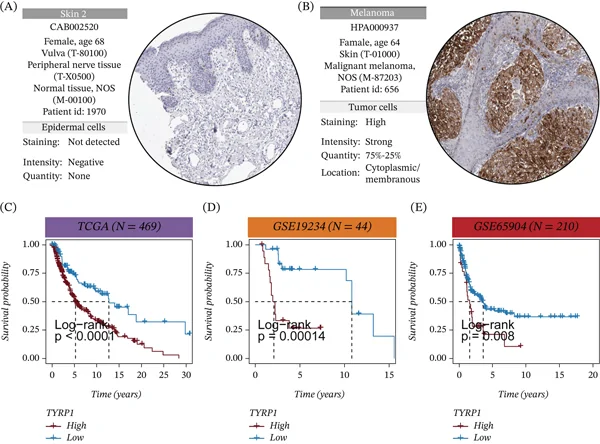
Protein expression and multicohort prognostic validation of TYRP1. (A) Representative IHC image of TYRP1 in normal vulvar skin tissue from the HPA database (CAB002520; female, age 68). TYRP1 staining is not detected in epidermal cells, with negative intensity. (B) Representative IHC image of TYRP1 in malignant melanoma tissue from the HPA database (HPA000937; female, age 64). Tumor cells display strong positive staining with cytoplasmic and membranous localization. (C–E) Kaplan–Meier survival curves for patients stratified by high (red) versus low (blue) TYRP1 expression in three independent cohorts: TCGA (*N* = 469), GSE19234 (*N* = 44), and GSE65904 (*N* = 210). Between‐group differences were assessed by the log‐rank test; *p* values are indicated within each panel. Dashed lines denote 50% survival probability. Source and figure preparation: The immunohistochemistry images in Panels A and B were obtained from the Human Protein Atlas (HPA; TYRP1 entry: https://www.proteinatlas.org/ENSG00000107165-TYRP1). Panel A corresponds to antibody CAB002520 and patient ID 1970; Panel B corresponds to antibody HPA000937 and patient ID 656. These images were generated by HPA and incorporated into the composite figure by the authors. Attribution: Human Protein Atlas, https://www.proteinatlas.org; licensed under the Creative Commons Attribution 4.0 International License (https://creativecommons.org/licenses/by/4.0/). Panels C–E were created by the authors specifically for this manuscript using R Version 4.4.2, based on data from The Cancer Genome Atlas Skin Cutaneous Melanoma project (TCGA‐SKCM), accessed through the Genomic Data Commons (GDC; https://portal.gdc.cancer.gov/projects/TCGA-SKCM) [22, 23], and GEO datasets GSE19234 (https://www.ncbi.nlm.nih.gov/geo/query/acc.cgi?acc=GSE19234) and GSE65904 (https://www.ncbi.nlm.nih.gov/geo/query/acc.cgi?acc=GSE65904) [20, 21].

## 4. Discussion

This study establishes an integrated research framework that proceeds from single‐cell transcriptomic heterogeneity through multicohort prognostic validation to functional dissection of the immune microenvironment, thereby providing a new molecular tool for precision prognostic stratification in melanoma. Specifically, the workflow proceeded from pseudotime‐resolved malignant‐cell states to identification of dynamically expressed genes, multicohort univariate Cox screening, and LASSO‐Cox reduction to a seven‐gene score, distinguishing this feature‐origin strategy from purely data‐driven machine‐learning signatures.

The tumor cell state evolution revealed by pseudotime trajectory analysis aligns mechanistically with a central tenet of melanoma phenotypic plasticity. The principal trajectory—originating from the melanocytic state, proceeding through neural crest‐like dedifferentiation, and terminating in an MHC‐II–high immunomodulatory endpoint—suggests a deep coupling between the progressive loss of differentiation and the acquisition of immune‐evasive capacity, consistent with the recently proposed model in which phenotypic plasticity drives adaptive immune resistance [25]. The stress/transitional state exhibits the broadest pseudotime distribution, positioning it as a hub node connecting multiple cell‐state pathways rather than an intermediate stop along a single linear route—implying that interventions targeting this state may hold broad‐spectrum potential for blocking plastic transitions. The proliferating, IFN‐stimulated, and invasive states cluster independently in pseudotime, suggesting that their emergence is driven by specific microenvironmental cues rather than sequential progression along the main differentiation axis; this “trunk‐and‐branch” topology may constitute the cellular basis underlying the heterogeneous responses of individual patients to immunotherapy and targeted therapy [26].

The seven‐gene prognostic model, built from the pseudotime‐dynamic gene repertoire, derives its core value from the fact that each constituent gene carries a well‐defined immunobiological rationale rather than emerging as a product of purely statistical selection. GBP2 and GBP4 belong to the interferon‐inducible GTPase family; their elevated expression not only signals sustained IFN‐*γ* signaling input but is also closely linked to inflammasome activation and enhanced MHC‐I presentation, and their enrichment in low‐risk patients reflects an actively engaged state of immune surveillance [27]. CIITA, the master transcriptional regulator of MHC‐II, confers upon tumor cells the capacity to directly participate in antigen presentation, supporting an emerging paradigm in which tumor cells noncanonically activate CD4^+^ T cells to shape adaptive immunity [28]. By contrast, the upregulation of TSC22D3 (GILZ) in high‐risk patients warrants particular attention—as a core effector of glucocorticoid signaling, it can broadly suppress T cell activation via inhibition of the NF‐*κ*B and AP‐1 pathways, constituting an endogenous, checkpoint‐independent immunosuppressive mechanism [29]. TNFSF13B (BAFF) points to the functional status of B cells within the TME; recent studies indicate that activation of BAFF signaling in tumor‐draining lymph nodes is closely associated with the formation of tertiary lymphoid structures and antitumor humoral immunity [30]. The inclusion of ITGB4 suggests a structural association between the invasive phenotype and immune evasion, as integrin *β*4–mediated basement membrane anchoring signals can activate the PI3K‐AKT axis, thereby downregulating the surface expression of multiple immune recognition molecules on tumor cells [31]. The functional convergence of these seven genes indicates that what the model ultimately captures is the integrated state of tumor cell “immunological visibility.”

The strong concordance between this model and the TIME provides a solid biological foundation for its clinical application. The immune‐inflamed phenotype observed in low‐risk patients—characterized by enrichment of effector T cells and broad upregulation of immune checkpoint molecules (PD‐1, LAG‐3, TIGIT, HAVCR2)—does not represent simple immune activation, but rather a functional, exhaustion‐like state that remains reversible through PD‐1/PD‐L1 blockade, closely overlapping with established predictive signatures of ICB response. High‐risk patients, characterized by high tumor purity and dual depletion of stromal and immune cell infiltration, display systemic suppression across multiple steps of the cancer–immunity cycle governing T cell recruitment, recognition, and killing, suggesting that their resistance mechanisms likely extend beyond any single checkpoint molecule and may require combinatorial strategies to overcome. The absence of a linear correlation between risk score and TMB, together with the superior prognostic resolution achieved through their joint stratification, further supports the notion that these two dimensions capture distinct biological facets of tumor immune evasion—the former reflecting intrinsic tumor immune programming, the latter reflecting neoantigen load—and that their integration offers a more comprehensive framework for refining patient selection for ICB therapy [32].

As the sole risk gene in the model, TYRP1 confers additional translational value through its dual attributes. Consistent validation at both the protein and transcriptomic levels across multiple cohorts establishes it as an independent prognostic biomarker, whereas its selective high expression in immune‐excluded, high‐risk tumors precisely matches the target‐antigen criteria required for emerging CAR‐T cell therapies—namely, high tumor expression coupled with low normal‐tissue toxicity [10]. This finding suggests that TYRP1‐directed adoptive cell therapy may represent a promising complementary strategy for the high‐risk patient population defined by this model, who derive limited benefit from ICB.

This study has several inherent limitations. The current analysis relies on retrospective public datasets, and validation in prospective clinical cohorts remains necessary. The single‐cell data were derived from a specific patient population, and the generalizability of the cell‐state atlas warrants systematic evaluation across diverse ethnic backgrounds and disease stages. Furthermore, the causal relationships between gene modules and cell states require clarification through functional experiments. Future studies should focus on validating the predictive value of the risk score for ICB efficacy in prospective cohorts, exploring intervention strategies targeting the stress/transitional state, and systematically evaluating in vivo the synergistic potential of combining TYRP1‐directed therapy with immune checkpoint blockade.

## Funding

This study was supported by the Young Scientists Fund of the National Natural Science Foundation of China (82103281).

## Ethics Statement

As no new human participants, biological samples, or animal experiments were involved, this study did not require additional ethical approval or informed consent, in accordance with the data use policies of the original repositories.

## Conflicts of Interest

The authors declare no conflicts of interest.

## Data Availability

The single‐cell RNA‐seq dataset analyzed in this study is available in GEO under accession GSE277165. Bulk gene‐expression datasets used for prognostic model construction and validation are available under accessions GSE19234, GSE22153, and GSE65904 [20, 21]. TCGA‐SKCM expression, mutation, and clinical data were obtained from the GDC data portal (https://portal.gdc.cancer.gov/projects/TCGA-SKCM) [22, 23]. TYRP1 immunohistochemistry images were obtained from the Human Protein Atlas (https://www.proteinatlas.org/). Hallmark gene sets were obtained from MSigDB (https://www.gsea-msigdb.org/gsea/msigdb) [15–17]. Other data supporting the findings of this study are available from the corresponding author upon reasonable request.
